# Production and sensory analysis of grape flavoured beer by co-fermentation of an industrial and a genetically modified laboratory yeast strain

**DOI:** 10.1007/s00217-023-04274-1

**Published:** 2023-04-30

**Authors:** Jorg C. de Ruijter, Heikki Aisala, Iina Jokinen, Kristoffer Krogerus, Heiko Rischer, Mervi Toivari

**Affiliations:** Sustainable Products and Materials, VTT Technical Research Centre of Finland Ltd, Espoo, Uusimaa Finland

**Keywords:** Co-fermentation, *Saccharomyces cerevisiae*, Methyl anthranilate, Brewing, Flavour

## Abstract

The so-called “craft beer revolution” has increased the demand for new styles of beers, often with new ingredients like flavour extracts. In recent years, synthetic biology has realized the production of a plethora of plant secondary metabolites in microbial hosts, which could provide an alternative source for these compounds. In this study, we selected a in situ flavour production approach for grape flavour addition. We used an *O*-methyl anthranilate (OmANT) producing laboratory *Saccharomyces cerevisiae* strain in co-fermentations with an industrial beer yeast strain WLP644. The laboratory strain provided an ease of genetic manipulation and the desirable properties of the WLP644 strain were not modified in this approach. In shake flasks, a 10:90 ratio of the yeasts produced grape flavoured beer with the yeast produced flavour compound in a range normally used for flavoured beverages. Hopped and unhopped beers were analysed by VTT’s trained sensory panel and with olfactory GC–MS. OmANT was successfully detected from the beers as a floral odour and flavour. Moreover, no off-flavours were detected and aroma profiles outside the grape flavour were rather similar. These results indicate that the co-fermentation principle is a suitable approach to change the flavour profiles of beers with a simple yeast strain drop-in approach.

**Supplementary Information:**

The online version contains supplementary material available at 10.1007/s00217-023-04274-1.

## Introduction

While the bulk of the global beer market is still dominated by industrially produced lager beer, the number of breweries, brands, and new beer styles is constantly increasing [1]. A large amount of these new, smaller breweries have been at the forefront of the ‘craft beer revolution’ that has transformed the industry in the past decades. The demand from consumers for beer with novel and diverse flavours has led to the development of new beer styles and introduction of new ingredients to the brewing process [2-4]. The latter includes the addition of new flavour extracts to beer, for example from fruits and berries, which historically might have been limited to certain beer styles, such as lambics [5]. Brewers may also attempt to diversify their products by using different yeast strains, as these natively produce a wide range of flavour compounds [6].

With the development of modern molecular biology tools, synthetic biology, and an increased understanding of plant and microbial metabolism, there has been a trend towards the production of biobased chemicals and natural products in fungal production hosts [7, 8]. One focus area of this research is the production of natural flavour compounds. These compounds are traditionally extracted from their natural hosts, such as plants, for example in the mentioned flavour extracts for beers. These extracts are complex mixtures and single compounds often exist at very minute amounts compared to the total biomass. Additionally, the whole process is often dependent on growing seasons of the plant host and thus sensitive to crop failures. In addition, limited availability of agricultural land space, and decreasing biodiversity lower the sustainability of the process. Therefore, microbial fermentation can be an alternative, sustainable source of these compounds, as no large fields would be needed for the production and production would be more predictable [9]. However, heterologous implementation of the production pathways for plant-derived compounds is not straightforward, often requiring a precise balance of the expression of multiple enzymes. Moreover, frequently more in-depth host genome engineering is necessary to increase the levels of necessary precursor metabolites or cofactors that are not readily available in the engineered microbial system [10]. Examples of flavour compounds produced in yeasts are the primary aroma compounds of vanilla, cinnamon, and raspberries, vanillin [11, 12], cinnamaldehyde [13], and raspberry ketone [14], respectively. And although some of these compounds are currently produced at rather low levels, the Swiss biotechnology company Evolva offers the commercial microbial production of valencene and nootkatone (important flavour components of orange peel and grapefruit, respectively).

Most of these production strains are based on laboratory yeast strains, which are very suitable for genetic engineering, but they might not have desirable properties for beverage fermentation. The different yeast strains used in beverage fermentations have been selected for their robustness and versatility in the production of desirable flavour compounds, but they might not be very suitable for genomic engineering due to their robust industrial characteristics. These yeasts are typically aneuploid with an average chromosome copy number of four [15], which hampers the modification of many different loci. Beer yeasts are also typically sterile, which also makes crossbreeding of such strains challenging. Additionally, the changes involved might affect the metabolism of the industrial yeast strain that relate to the flavour profiles produced in the beers. Nevertheless, there are examples of the introduction of heterologous pathways into industrially relevant beer and wine yeasts for the production of heterologous aroma compounds. Examples include the production of two monoterpene alcohols found in hops, linalool and geraniol, during beer fermentation and the primary aroma compound in raspberries, raspberry ketone, produced during wine fermentation [16]. In addition, beer and wine yeast have been modified to enhance formation of natively produced compounds, such as 3-sulfanylhexan-1-ol and its corresponding acetate ester, which impart grapefruit and tropical fruit aromas, respectively [17, 18].

In a recent study, we engineered a laboratory *S. cerevisiae* strain for the production of *O*-methyl anthranilate (OmANT), a compound with grape flavour [19]. This compound is naturally found in grapes, and its chemically synthesized alternative has been used extensively for the flavouring of food and beverages [20, 21]. Due to the complexity of the pathway, implementing it into an industrial beer fermenting yeast would be laborious. However, the parental CEN.PK strains are also able to grow well under beer brewing conditions and can consume maltose and maltotriose in the wort directly [22]. Therefore, in this study, we employed a mixed fermentation strategy, where conditions for normal beer production were kept the same, except for the addition of the *O*-methyl anthranilate (OmANT) producing yeast to the inoculation biomass. We optimized the biomass ratio to get the desirable amount of the flavour compound produced in the beer and performed a sensory evaluation on the final product.

## Materials and methods

### Yeast strains and media

The OmANT-producing yeast strain H5626 is derived from the laboratory strain CEN.PK113-17A and its construction was described previously [19]. In short, the strain has a modified amino acid metabolism to increase anthranilate production and expresses the anthranilic acid methyltransferase 1 (MtAAMT1) gene from *Medicago truncatula* to produce OmANT. The conventional brewing strain used was WLP644 from White Labs. Strains for precultures were routinely grown in YP media (10 g L^−1^ yeast extract, 20 g L^−1^ Peptone), with 20 g L^−1^ glucose or 40 g L^−1^ maltose, for solid media this was supplemented with 20 g L^−1^ agar. Yeast precultures were carried out at 20 °C or 28 °C with 230 rpm shaking, main fermentations were carried out at 20 °C without aeration.

### Small scale flask fermentations

Small scale fermentations were carried out with 50 mL volumes in 250 mL shake flask with an airlock. Cultures were inoculated into a wort produced from barley malt at a total start OD_600_ of 1, and incubator temperature was set at 20 °C. For seven days a 1 mL culture supernatant sample was taken every 24 h, for HPLC and UPLC-MS analysis. Final fermentation samples were subjected to analysis using an Anton Paar density meter, as described below. HPLC was used to measure sugar concentrations and alcohol content, as described previously [23]. Compounds were separated with Fast Acid Analysis Column (100 × 7.8 mm, BioRad Laboratories, Helsinki, Finland) and Aminex HPX-87H organic acid analysis column (300 × 7.8 mm, BioRad Laboratories, Helsinki, Finland) connected to Waters 2690 separation module. Peaks were detected with Waters 2414 differential refractometer (Waters, Milford, MA). The column was eluted with 5 mM H_2_SO_4_ at a 0.5 mL/min flow rate at 55 °C.

Samples for UPLC-MS were separated using an Atlantis Premier BEH C18 AX 1.7 µm, 2.1 mm X 100 mm column (Waters), kept at 60 °C. Flow rate of the Mobile phase A (1 mM ammonium formate in water, pH 3) and B (1 mM ammonium formate in 50% isopropanol:50% acetonitrile) was 0.350 mL/min. Following gradient program was used: 0 min: 100% A, 6 min: 100% B, 6.10 min 100% A, 10 min 100% A. The analysis was performed with anthranilate (Sigma, purity ≥ 98%) and OmANT (Sigma, purity ≥ 98%) as analytical standards for the identification and quantification of the products.

### 2 L-scale high gravity wort fermentations

Strains were characterized in fermentations performed in a 15°P wort at 20 °C. Fermentations were carried out in duplicate 2-L cylindroconical stainless steel fermenting vessels, containing 1.5 L of wort medium. Two different 15°P worts (with and without hops in the boil) were produced at the VTT Pilot Brewery from barley malt (the sugar composition of the worts was 69 g of maltose, 17.4 g of maltotriose, 15.1 g of glucose, and 5.0 g of fructose per litre). Yeast was inoculated at a rate of 15 × 10^6^ viable cells mL^−1^ in total. The wort was oxygenated to 10 mg L^−1^ prior to pitching (oxygen indicator model 26,073 and sensor 21,158; Orbisphere Laboratories, Switzerland). The fermentations were carried out at 20 °C until the alcohol level stabilized, or for a maximum of 9 days. Wort samples were drawn regularly from the fermentation vessels aseptically and placed directly on ice, after which the yeast was separated from the fermenting wort by centrifugation (9000 × g, 10 min, 1 °C). Samples for yeast-derived flavour compound analysis were drawn from the beer when fermentations were ended.

The alcohol level (% vol/vol) of samples was determined from the centrifuged and degassed fermentation samples using an Anton Paar density meter DMA 5000 M with Alcolyzer beer ME and pH ME modules (Anton Paar GmbH, Austria). OmANT levels were measured as described above.

### Sensory panel sample preparation

After the 2 L fermentations were finished, the beers were centrifuged for 30 min at 4000 rpm and clarified supernatants were sterile filtered using a 500 mL bottle-top vacuum filtration system with a 0.42 µm SFCA filter. After measurement of the OmANT levels, the respective co-inoculated fermentations were adjusted with their corresponding control fermentation so that the final OmANT concentration was approximately 10 mg/L. A total of five beer samples was prepared; unhopped wort control, unhopped wort spiked with food grade OmANT, unhopped wort with microbially produced OmANT, hopped wort control, and beer 5: hopped wort with microbially produced OmANT. A reference sample for OmANT intensity was prepared by adding food grade OmANT to water to a final concentration of 10 mg/L.

### Sensory profiling

The sensory profiles of the beer samples were analyzed by ten assessors of VTT’s trained food and beverage sensory panel with generic descriptive analysis. The sensory evaluation was done in VTT’s ISO-8589 sensory evaluation laboratory. An application regarding the sensory evaluation was made to VTT’s internal ethical committee. The risk mitigation strategies for the panel included sterile filtering the produced beer samples, analyzing the microbiological quality of the samples prior to the evaluation, following a taste-and-spit assay, making specific COVID-19 precautions, and requesting prior written informed consents (with exclusion criteria) from the assessors.

The base attribute list for the beer samples was formulated by four panel members in a consensus tasting session. Previous sensory profiles of VTT’s beers were used as templates [24, 25]. This lexicon was introduced to the whole panel in panel training (divided in two groups). Additionally, two of the ten assessors had separate training and evaluation sessions due to time schedules. During training, the attributes names and descriptions were refined, the variation in intensity was discussed by evaluation of extreme samples, and the reference products intensities were tied to the 0–10 line scale.

The resulting sensory lexicon had six odour attributes, four taste or flavour attributes, and two chemesthesis or mouthfeel parameters. The profile also had five reference products for the attributes (please see Table S1 in the Supplementary Material for the list of attributes and the reference products). The samples were presented monadically in a balanced complete block design using Latin squares serving order randomization. Two replicate evaluations were made. For the evaluation, 30 ml of each sample was served in black beer glasses covered with lids. The samples were marked with 3-digit codes and served in ambient temperature. The sensory data was collected using EyeQuestion version 5.0.7.15 (with EyeOpenR Data Analysis) by EyeQuestion Software (Elst, the Netherlands) and Qi Statistic Ltd. (West Malling, UK).

### Analysis of volatile compounds

The volatile compounds of the beers adjusted for sensory profiling were analysed with a method adapted from one reported by Krogerus et al. [24]. Briefly, the headspace volatiles were first extracted by solid phase microextraction (SPME) with 2 cm 50/30 μm divinylbenzene/carboxen/ poly(dimethylsiloxane) (DVB/CAR/PDMS) fiber (Stableflex, 23 Ga, Autosampler) (Supelco, Bellafonte, PA) at 70 ℃ for 30 min. A DB-FFAP column (25 m × 200 µm × 0,33 µm) was used to separate the compounds on an Agilent 7890A + 5975C GC–MS instrument (Agilent Technologies, CA, US). Scan range of 25–500 amu was used. 3-octanol and 3,4-dimethylphenol were used as internal standards in isomolar amounts. The peak areas were normalized to internal standard peak areas.

### Olfactory GC–MS (GC-O)

The HS–SPME–GC–MS/O analysis of the beers adjusted for sensory profiling was applied from the protocol by Thompson-Witrick et al. [26]. Samples were stored in a dark storage in 4 °C until GC-O analysis.

Volatile compounds were extracted by SPME with a 2 cm DVB/CAR/PDMS fiber. The SPME parameters were adapted from Witrick et al. [27]. 1.0 mL of each beer sample were incubated using the autosampler (Combi PAL, PAL System, CTC Analytics AG, Zwingen, Switzerland), at 40 °C for 30 min. Each beer extract was injected in splitless mode to a VF-WAXms column (60 m × 0.25 mm × 0.5 µm) (Agilent Technologies, CA, US) and volatiles were desorbed for 6 min at 250 °C. The GCMS-O system consisted of a 6890N GC (Agilent Technologies, CA, US) equipped with a mass detector (5973-Network), and a sniffing port ODP4 (Gerstel, Baltimore, MD) supplied with humidified air. The GC effluent was split 1:1 between the mass detector and sniffing port. The flow rate of the helium gas was set to 2.0 ml/min. The GC oven program was as follows: hold at 50 °C for 1 min, from 50 to 240 °C at 12 °C min^−1^, and held at 240 °C for 8 min. MS scan range was set to 25–600 amu, at 2.0 scans/s. Temperature of the MS detector was set at 230 °C. The volatiles were identified based on (a) NIST library (vs2.3, 2017), and (b) a linear retention index based on a hydrocarbon mixture (C7-C30 saturated alkanes, Supelco, Bellafonte, PA) which were compared to values in the literature and (c) odour properties. In addition, methyl anthranilate was identified based on the authentic standard compound.

GC-O evaluation was performed with the detection frequency method with panel of four (three males and one female). All panellists were previously trained in odour recognition and sensory evaluation techniques and had experience in GC-O. The panellists were asked to describe the odour and to record the duration of each odorant. Detection of an odour at the sniffing port by three or more assessors were considered significant.

### Statistical analysis

The sensory data were examined with a two-way mixed model analysis of variance (ANOVA), with samples as the fixed factor and assessors as the random factor. Tukey’s HSD was used as the post hoc test. Principal component analysis was performed on consensus data (averaged over replicates and assessors) with autoscaled data.

## Results and discussion

### Strain profiles and co-culture design

Previously, we have engineered a platform laboratory *S. cerevisiae* strain (derived from haploid CEN.PK113-1A) that produces high levels of anthranilate, with concentrations measured well above 500 mg/L. Introduction of the *MtAAMT1* gene, encoding the *M. truncatula* anthranilic acid methyltransferase 1 (creating strain H5626), led to production the grape flavour compound *O*-methyl anthranilate, upwards of 400 mg/L in rich media [19]. The detection threshold for OmANT was measured at around 8 µg/L in water, and 45–89.4 µg/L in wines [28]. The distinct sweet, fruity smell of these cultivations and the fact that synthetic OmANT is well used in flavoured beverages made us consider employing this flavour for beer fermentation purposes.

The OmANT-producing strain has undergone extensive genetic manipulation, including the deletion of one gene, duplication of three native genes, switching of eight promoters, and integration of one heterologous gene. Therefore, we decided to circumvent complex genetic engineering techniques for an industrial brewing yeast. Instead, we explored whether “co-fermentation” using different ratios of the OmANT-producing laboratory strain together with the industrial beer yeast strain WLP644 could be used to produce beer with an enhanced grape flavour. This setup would benefit from the general flavour profile produced by the industrial yeast strain, with the addition of the specific flavour compound produced by the CEN.PK strain. This all without the need of changing the industrial nor process parameters. The range of concentration of OmANT in commercial grape juices and sodas was measured to be 1.1–16.6 mg/L [21, 29]. Because our production strains H5626 produces about 30 times more than that in shake flask cultivations, we expected that only a relatively small proportion of the inoculum would need to be the laboratory strain. Therefore, the overall background flavour profile produced by the WLP644 strain would not be affected significantly with possible off flavours from the CEN.PK strain.

### Small-scale cultivations

**Fig. 1:**
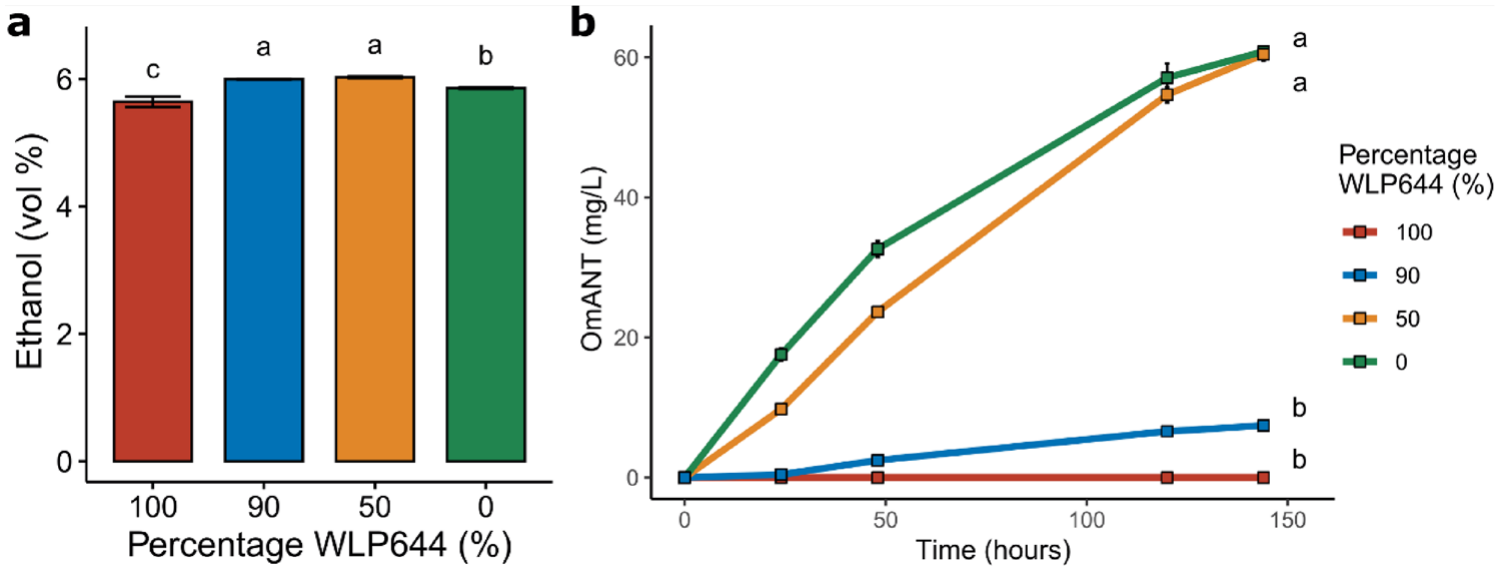
Ethanol production (**A**) and OmANT levels (**B**) of the small-scale cultivations. All data presented is the average of three replicate shake flask fermentations, with error bars representing the standard deviation

We cultivated mixtures of WLP644 and H5626 in shake flasks, using a wort made from barley malt, selecting strain ratios of 100:0, 90:10, 50:50, 10:90 and 0:100. Cultivations were run at 20 °C without shaking and samples were taken every 24 h for analysis of OmANT production. After seven days the cultivations were ended, and degassed samples were analysed for alcohol concentration and residual sugar. The cultivations that included the OmANT-producing strain had slightly higher ethanol concentrations and thus also slightly lower residual sugar concentration compared to the pure WLP644 cultivation (Fig. 1A). The final pH of the different ratios of co-inoculations was similar and in the range of 4.30–4.46. This is probably related to the efficient maltose and maltotriose consumption of the CEN.PK-based strain. As expected, OmANT production increased with an increased ratio of the strain H5626 in the fermentation (Fig. 1B). Interestingly, using 50 or 100% of the H5626 strain led to a rather similar flavour production profile with almost identical final concentrations of OmANT. However, the concentrations were about four times higher than the highest reported for beverages [20, 21]. Indeed, the flavour and odour of the OmANT in these beers was very pungent and overpowering. Moreover, the beer fermented solely with H5626 lacked the distinct beer flavour profile created by the WLP644 strain (delicate mango and pineapple characteristics, as described by the supplier). The fermentation with 10% H5626 showed an almost linear OmANT production over time after the first 24 h. At the end of the fermentation, the cultivation contained 7.42 ± 0.55 mg/L OmANT and had a pleasant subtle grape smell. The OmANT concentration fits very well within the range of the concentrations reported before for commonly available grape sodas. To provide enough flavoured beers for sensory analysis, we used these conditions for further production in scale-up beer fermentations in VTTs pilot brewery.

### 2 L scale-up fermentations

**Fig. 2:**
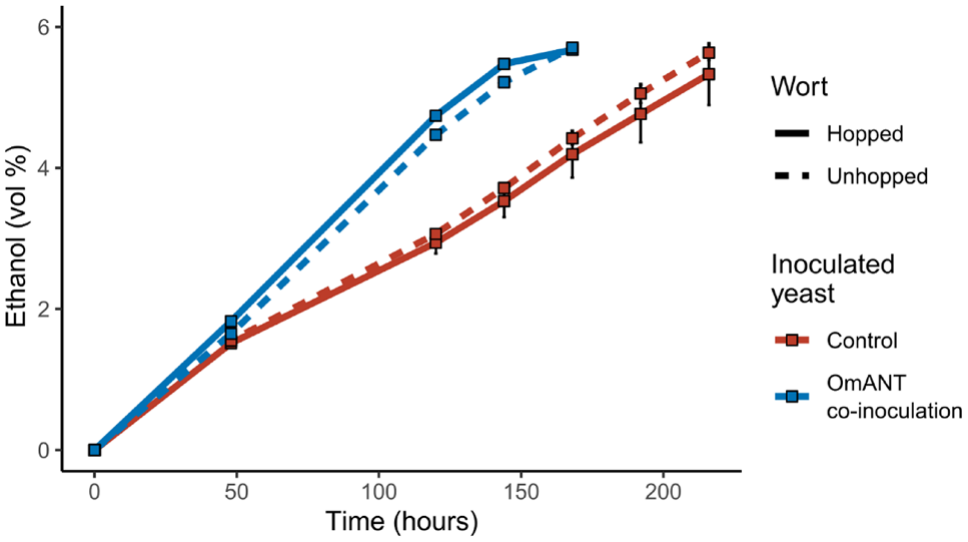
Ethanol production over time of the different fermentations at the 2L scale. Data shown is average of two fermentations, with error bars showing the range of the data

Fermentations were carried out in duplicate 2 L cylindroconical stainless steel fermenting vessels at the VTT Pilot Brewery. We used both wort with and without hops added during the boil, to ensure flavour from the hops did not mask that from OmANT. Both worts were inoculated either with WLP644 by itself (control) or a mixture of WLP644 and H5626. Yeast was inoculated at a rate of 15 × 10^6^ viable cells mL^−1^, with a ratio of 90:10 WLP644:H5626 for the mixed fermentations. During the fermentations, regular wort samples were taken and analysed for ethanol production and residual sugar (Fig. 2). After 7 days the ethanol levels in the fermentations with 90:10 WLP644:H5626 had started to stabilise, and those fermentations were ended. The control WLP644 fermentations were fermenting slower and were continued for another 48 h until they reached comparable ethanol levels. All final pH levels were similar and in the range of 4.31–4.40. The OmANT levels were 16.29 ± 1.23 mg/L for the unhopped wort and 15.99 ± 0.13 mg/L for the hopped wort. As we aimed for slightly lower concentrations of OmANT to avoid a too intense grape flavour we adjusted the OmANT concentrations to 10 mg/L using the 100% WLP644 fermentations.

To gain some insights in possible differences between the beers we measured volatile compounds in the headspace of the final, adjusted beers using GC–MS (Figure S1). The aroma profiles measured were similar, but with some slight differences. The control beers contained higher amounts of ethyl acetate and 3-methyl butyl acetate (1.2- and 1.5-fold, respectively), which are important beer esters produced by the WLP644 strain [30]. The beers with the H5626 strain present in the fermentation contained higher amounts of 2-phenylethanol and 2-phenylethylacetate (1.8- and 2.2-fold, respectively), and naturally the OmANT produced by this strain.

### Sensory profiling

**Fig. 3:**
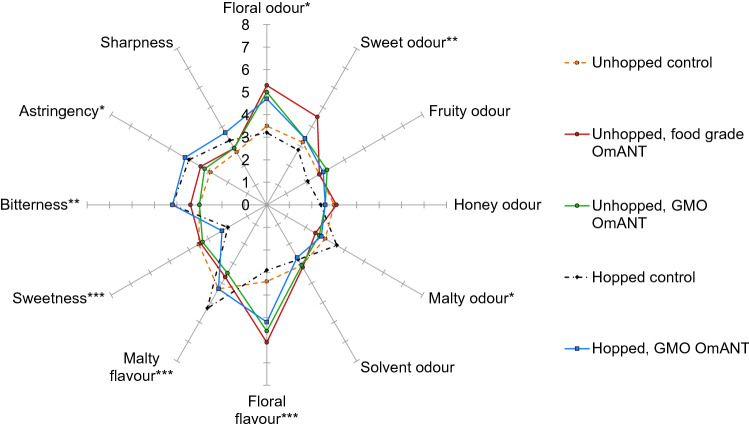
Spider plot of the sensory profiling of the five beers. Attributes with asterisks have statistically significant differences between samples in the two-way mixed model ANOVA (*: *p* < 0.05, **: *p* < 0.01, ***: *p* < 0.005)

The four produced beers were subjected to sensory analysis, with the addition of the unhopped control beer spiked with 10 mg/L food grade OmANT. The five studied beers were profiled by ten trained assessors with 12 sensory attributes (Table S1). All attributes were detected in each sample but with different intensities depending on the sample (Fig. 3, Table S2, Figure S2). The sampled beers were characterized by moderate fruity, malty, honey, and solvent odours. The largest difference between samples was in the added hops (separation in principal component 1 in Figure S2). The samples with hopped wort had lower sweetness and more intense bitterness, along with a higher malty flavour and astringency in some samples.

The presence of OmANT resulted in strong, specific changes to the odour and flavour profile (separation in principal component 2 in Figure S2): it consistently intensified the floral odour and flavour in the beers (Fig. 3, Table S2). This effect was observed both when produced by the modified yeast or when added as a pure compound and was observed in both base worts. When added as a pure compound, the resulting beer was also characterised with a sweeter odour. This difference between supplemented food grade OmANT and yeast-produced OmANT is likely related to small changes in the sample preparation timeline for the beer samples. The supplemented beers were prepared closer before evaluation and thus the OmANT had less time to be released from the matrix and was more perceivable for the panellists.

### Odour active compounds by GC-O

The odour active volatiles of the unhopped and hopped co-inoculated OmANT beers (adjusted for sensory profiling) were identified with detection frequency method by GC-O with four trained panellists. Fifteen compounds with a Nasal Impact Factor (NIF) above 50% were detected in the unhopped OmANT beer, whereas eighteen were detected in the hopped OmANT beer (Table 1). Identification was obtained by comparing the calculated linear retention index (LRI) values, mass spectra, and odour descriptors to those of pure compounds. In general, the observed odour compounds were described as floral, fruity, and sweet odours, but also as chemical-like odours.

**Table 1:** Odour active compounds of unhopped and hopped GMO OmANT beers

LRI^a^ (VF-WAX)	Compound	Odour description^b^	LRI (database)	Identification	NIF^c^ (%)	
					Unhopped OmANT	Hopped OmANT
985	Propyl acetate	Chemical, fruity, permanent marker	953	O, MS, RI	50	50
1053	Ethyl butyrate	Permanent marker	1028–1035	O, MS, RI	50	0
1145	Isoamyl acetate	Pear, banana, candy, floral	1122	O, MS, RI	100	75
1207	Methyl hexanoate	difficult to describe	1184	O, MS, RI	50	50
1226	Isoamyl alcohol	chocolate, butyric acid, unpleasant	1209–1226	O, MS, RI	75	0
1252	Ethyl hexanoate	Fruity, candy	1226–1233	O, MS, RI	75	75
1383	Methyl 3-aminobenzoate (tentative) *Main ions: 64, 92, 119, 151*	Sweet, essence, perfume		MS	50	50
1451	Ethyl octanoate	Glue, difficult to describe	1435	O, MS, RI	50	50
1506	Furfural	rye bread, mushroom, yeast	1462–1493	O, MS, RI	0	75
1702	Unknown	Mushroom, unpleasant			50	50
1868	Phenethyl acetate	beer, rose hip, fruity, clove, currant	1813	O, MS, RI	75	75
1895	Unknown	difficult to describe			0	50
1965	Phenethyl alcohol	Beer like, floral, pollen	1903–1956	O, MS, RI	0	75
1975	Lauryl alcohol	Fragrance, beer like, rose	1961–1966	O, MS, RI	50	0
2086	Unknown	Cotton candy, burned sugar, sweet			75	100
2125	Unknown	Sweet, musty, roasted sugar			75	50
2286	Unknown	Medicine, ibuprofen			75	50
2298	Unknown	Bread like, sweet			0	50
2309	Unknown	Sweet, essence			0	50
2322	Methyl anthranilate (OmANT)	Floral, perfume, grape essence, candy	2232	O, MS,RI,std	100	75
2366	unknown	Medicine, ibuprofen			0	50

^a^Linear retention index

^b^Odour description by panellists

^c^Nasal Impact Factor

Majority of the observed odour active compounds have been previously identified in various types of beer [26, 27, 31, 32]. Only propyl acetate, methyl hexanoate, and methyl anthranilate (OMAnt) have not been described in the previous literature. Isoamyl acetate, phenethyl acetate, ethyl hexanoate, and ethyl octanoate that were observed in both beers are considered as esters that have a major contribution to the beer aromatic profile as reviewed by Witrick et al. [27]. These compounds all had equal or higher NIF values than 50%. In addition, unknown compound with LRI 2086 and OmANT were identified as other key odour active compounds (NIF% equal or more than 75%) of unhopped and hopped OmANT beers. OmANT was observed in both beer samples by the panellists and was described as “floral, grape essence, perfume, candy”. Interestingly, an odour active compound with similar description was also observed at LRI 1383 and was considered likely to be an isomer of OmANT as the MS fragmentation pattern of this compound is almost identical and has the same molecular mass as OmANT. Based on the fragmentation pattern and main ions, this compound was tentatively identified as methyl 3-aminobenzoate. Eight odour active compounds were detected by the panel in both OmANT beer samples that were not identified based on mass spectrum and are referred as unknown compounds.

Addition of hops is known to significantly affect the odour active compounds in beer [31]. Although the unhopped and hopped OmANT beers were very similar in their odour active volatile profiles, also differences were observed. Ethyl butyrate (permanent marker), isoamyl alcohol (chocolate, butyric acid, unpleasant), and lauryl alcohol (beer like, rose, fragrance) were only observed in the unhopped OmANT beer. Furfural (rye bread, mushroom, yeast) and phenethyl alcohol (floral, beer like, pollen) were only observed in the hopped OmANT beer. Furthermore, majority of the unknown compounds were observed only in the hopped OmANT beer. OmANT was observed more profoundly in unhopped OmANT beer, with NIF value 100% compared to the NIF value of 75% in hopped OmANT beer.

The sensory profiling demonstrated that the current mixed fermentation approach affected only the desired attributes, as no unexpected or undesirable flavour traits were reported. H5626 did not produce any phenolic off-flavours, which are common among non-brewing *S. cerevisiae* strains, as the host strain contains a non-functional *PAD1* gene [33]. Additionally, the GC–MS results showed that even though there were differences in the volatile compound profiles between the beers with and without the engineered yeast, they were so small that the sensory panellists were not able to observe these differences. This enables brewers to tailor their products with specific aroma and flavour properties based on the selected strains.

The mixed fermentation approach is a straightforward, easy to use approach to change the flavour profiles of beers. In the example case here, we had one strain producing one flavour compound. However, multiple related flavour compounds could be produced by one strain, or one could aim to add multiple strains producing different compounds for a more complex change of the overall flavour bouquet. In addition, many commercial wine and beer brewing yeast strains have been found to be hybrids from *S. cerevisiae* strains with other (related) yeast species [34]. Therefore, it might also be possible to introduce the heterologous pathways into the industrial beer yeast strains through breeding or hybridization. This could then work as an alternative to directly modifying the genome of the brewing strains, however, such an approach would not work well for production pathways requiring multiple deletions of native genes.

More complex is the approach to modify the industrial brewing strain to produce the desired compounds in a single strain fermentation. Denby and colleagues used a combinatorial approach in their paper to produce two major hops compounds [16]**.** For this they tested the four genes of the pathway in 18 different combinations to find the optimal conditions for integration into the brewer’s yeast genome with an optimized CRISPR-Cas9 strategy. However, they reported a reduced fermentation capacity in some of these strains, even though they were able to superimpose the production pathway on the native metabolome. In the case that we present here, the production of OmANT, there has been an extensive modification of the core metabolism to increase product flow through the shikimate pathway towards anthranilate [19]. This would require many targeted integration and deletion steps in the polyploid industrial strains, and they would need to be homozygous for them to fulfil their function in the pathway. Moreover, there is a chance that this would influence the fermentation capacity and/or flavour profile of the yeast strain. Kuivanen et al. did report growth deficiencies on different media that would make this a probable outcome. Nevertheless, here, the H5626 strain was able to ferment wort efficiently, even more so than the WLP644 brewing strain.

While traditional non-GM strain improvement strategies, such as breeding and adaptive evolution, can be used to enhance properties of yeast [35, 36], it is unfeasible to introduce completely novel properties. Hence, to truly expand the diversity of strains available for beverage fermentations, the use of genetic engineering might be a necessity. Recent population genomics studies of *S. cerevisiae* have revealed that industrially used strains are genetically similar depending to niche (e.g. brewing, wine, dairy and sake), and that many strains from different suppliers were found to be almost genetically identical [15, 37]. Therefore, it has been suggested that breeding development of new strains with better desirable traits would be difficult and genetic engineering could be necessary. Currently, the first genetically modified wine and beer yeast strains have been approved for use in food production by the FDA.

The vast majority of the beer produced today is fermented with a single pure yeast strain. However, a small segment of speciality beers, such as maltøl, lambics, Berliner Weiße, and other sour ales, utilize mixed cultures to produce their signature flavours. In such mixed fermentations, the different strains contribute different functionality and flavour. The use of non-conventional yeasts for bioflavouring together with brewing yeast in mixed fermentations has been shown to introduce a wide diversity of flavours to beer [38-40]. Similarly, lactic acid bacteria can also contribute a range of flavour compounds through mixed fermentation [41]. Because brewer’s wort and beer are not an optimal growth media for most non-conventional yeasts, they typically have to be inoculated prior to the brewer’s yeast to achieve maximum flavour contribution [38, 42]. Here, bioflavouring was instead conducted by an engineered *S. cerevisiae* strain. Compared to the mixing of completely different species, it is here expected that the two strains would have more similar metabolism and performance in the wort. Indeed, the engineered H5626 even outperformed the WLP644 strain in regard to fermentation and was therefore well suited for a co-inoculated fermentation. A similar approach of blending a *S. cerevisiae* wine strain with an engineered strain to produce geraniol was used to produce wine with more restrained terpene levels, compared to those produced by the engineered strain alone [43]. Nevertheless, from an industrial process point-of-view, the use of multiple strains is challenging. Firstly, brewers typically reuse yeast for multiple consecutive fermentations, and the relative abundance between strains will change during fermentation. For a similar reason, the propagation of starter cultures would need to be carried out separately. Despite this, mixed fermentations are, as described above, already used for production, and the strategy used here could also be applied for further testing and product development.

## Supplementary Information

Below is the link to the electronic supplementary material.

Supplementary file1 (DOCX 542 KB)

## Data Availability

All data included in this manuscript are available upon request by contacting with the corresponding author.
