# Supplementary material for: Production and sensory analysis of grape flavoured beer by co-fermentation of an industrial and a genetically modified laboratory yeast strain
Source: Eur Food Res Technol. 2023 Apr 30:1–10. Online ahead of print. doi: 10.1007/s00217-023-04274-1 (PMC10148978; doi:10.1007/s00217-023-04274-1)
Supplement: Supplementary file 1 — Supplementary file1 (DOCX 542 KB) [file 217_2023_4274_MOESM1_ESM.docx]

Production and sensory analysis of grape flavoured beer by co-fermentation of an industrial and a genetically modified laboratory yeast strain.

Jorg C. de Ruijter*, Heikki Aisala, Iina Jokinen, Kristoffer Krogerus, Heiko Rischer, and Mervi Toivari

VTT Technical Research Centre of Finland Ltd, Sustainable Products and Materials, Espoo, Uusimaa, Finland

* Corresponding author: [jorg.deruijter@vtt.fi](mailto:jorg.deruijter@vtt.fi)

**Supplementary materials**

Table S1. Sensory attributes, definitions, reference products and their bound intensities on a 0-10 line scale used in the sensory profiling of the five beers.

| # | Attribute | Definition | Reference | Intensity |
| --- | --- | --- | --- | --- |
| Odour | | | | |
| 1 | Floral odour | Floral, perfume-like or odour similar to candy or chewing gum essence | Methyl anthranilate in water, 30 ml | 7 |
| 2 | Sweet odour |  | - |  |
| 3 | Fruity odour | Fruitiness similar to apples or pears | - |  |
| 4 | Honey odour |  | Pure honey  (Kesko Oyj, Finland),  6 g | 10 |
| 5 | Malty odour | Beer malts | Pilsner malt, 45 C heating cabinet for 15 min, 15 ml |  |
| 6 | Solvent odour | Similar to solvents such as nail polish (acetone) | - |  |
| Flavour | | | | |
| 7 | Floral flavour | Floral, perfume-like or flavour similar to candy or chewing gum essence | Methyl anthranilate in water, 30 ml | 9 |
| 8 | Malty flavour | Beer malts | Pilsner malt, 45 C heating cabinet for 15 min, 15 ml |  |
| 9 | Sweetness |  | Sucrose, 3.0% solution | 8 |
| 10 | Bitterness | Bitter sensation that is more prominent at the end of the tasting | Caffeine, 0.07% solution | 8 |
| Chemesthesis | | | | |
| 11 | Astringency | Mouthdrying sensation | - |  |
| 12 | Sharpness | Prickly or fizzy sensation perceived after some seconds of moving the sample around the mouth | - |  |

Table S2. Sensory profiling results of the five beers, standard deviations of sensory attributes are denoted in brackets. ANOVA p values point to the two-way mixed model, with samples as a fixed factor and assessors as a random factor. The different letters in each attribute signify statistically significant differences between samples in Tukey’s HSD post hoc test.

| **Attribute** | Anova p | **Unhopped control** | | | **Unhopped, food grade OmANT** | | | **Unhopped, GMO OmANT** | | | **Hopped control** | | | **Hopped, GMO OmANT** | | |
| --- | --- | --- | --- | --- | --- | --- | --- | --- | --- | --- | --- | --- | --- | --- | --- | --- |
| Floral odour | **0.015** | 3.5 | (2.5) | AB | 5.3 | (2.1) | A | 5.0 | (2.0) | AB | 3.2 | (2.6) | B | 4.7 | (2.5) | AB |
| Sweet odour | **0.002** | 3.2 | (1.4) | B | 4.5 | (1.7) | A | 3.4 | (1.7) | AB | 2.8 | (1.8) | B | 3.4 | (1.9) | AB |
| Fruity odour | *0.145* | 2.7 | (1.9) | A | 2.7 | (1.9) | A | 3.1 | (1.5) | A | 2.1 | (1.8) | A | 2.9 | (1.7) | A |
| Honey odour | *0.474* | 3.0 | (2.2) | A | 3.2 | (2.5) | A | 2.6 | (2.3) | A | 2.4 | (2.1) | A | 2.6 | (2.2) | A |
| Malty odour | **0.036** | 3.0 | (1.5) | AB | 2.5 | (1.2) | B | 2.7 | (1.3) | AB | 3.6 | (1.4) | A | 2.8 | (1.1) | AB |
| Solvent odour | *0.313* | 3.1 | (2.1) | A | 3.2 | (1.9) | A | 3.1 | (1.9) | A | 2.8 | (2.2) | A | 2.7 | (1.6) | A |
| Floral flavour | **<0.001** | 3.4 | (2.6) | BC | 6.1 | (2.5) | A | 5.6 | (2.7) | A | 2.9 | (2.9) | C | 5.2 | (2.8) | AB |
| Malty flavour | **<0.001** | 4.3 | (1.7) | AB | 3.7 | (1.4) | B | 3.5 | (1.6) | B | 5.3 | (1.6) | A | 4.3 | (1.5) | AB |
| Sweetness | **<0.001** | 3.5 | (2.1) | A | 3.4 | (2.1) | A | 3.3 | (1.9) | AB | 2.0 | (1.3) | C | 2.4 | (1.6) | BC |
| Bitterness | **0.003** | 3.0 | (1.7) | B | 3.4 | (1.9) | AB | 3.0 | (1.9) | B | 4.2 | (1.7) | A | 4.2 | (1.4) | A |
| Astringency | **0.028** | 2.9 | (1.6) | B | 3.4 | (1.5) | AB | 3.2 | (1.5) | AB | 4.0 | (1.8) | AB | 4.2 | (2.0) | A |
| Sharpness | *0.323* | 2.7 | (1.9) | A | 2.9 | (1.7) | A | 2.9 | (2.3) | A | 3.3 | (2.1) | A | 3.7 | (2.9) | A |

 
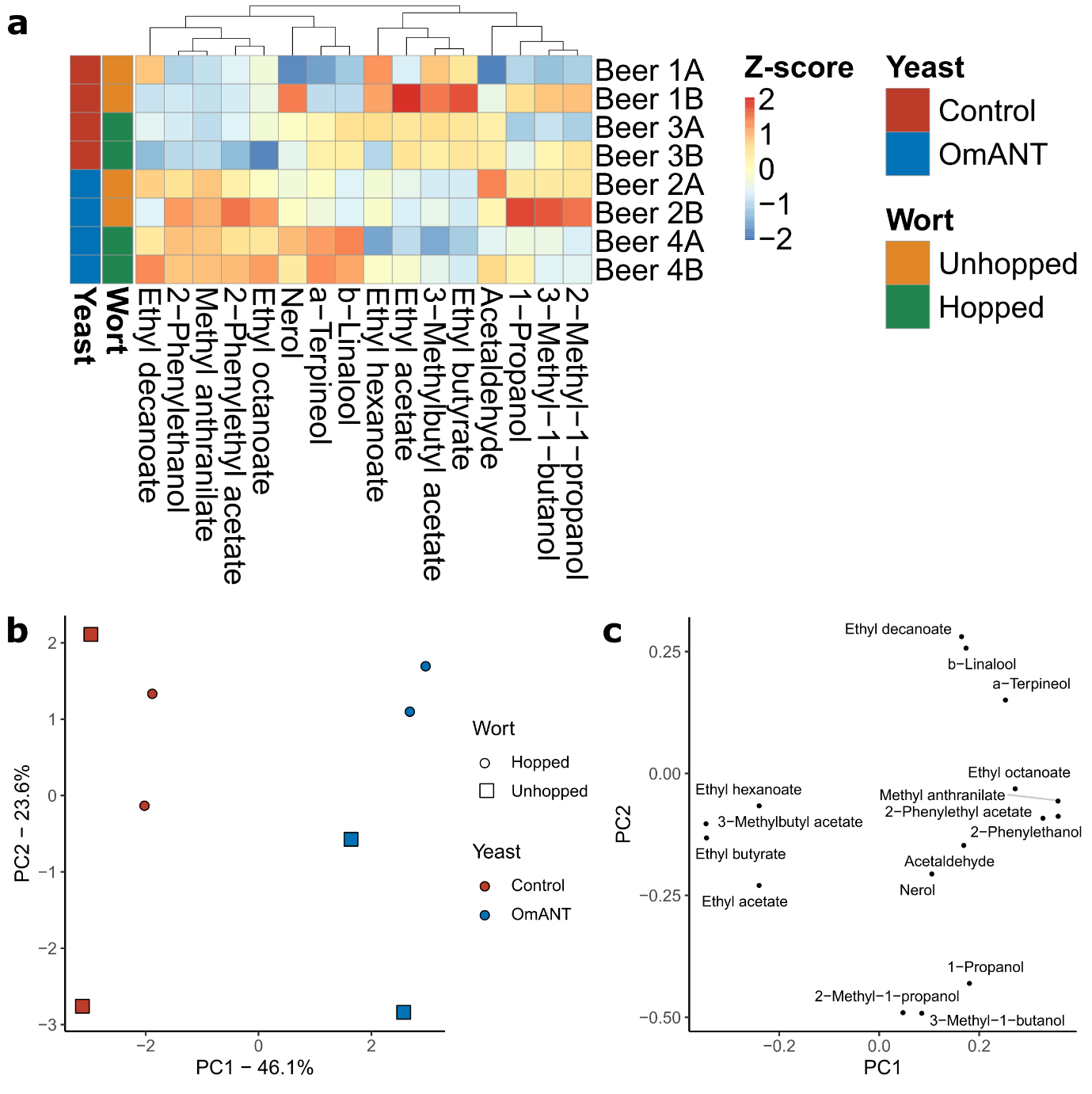


**Figure S1**. GC-MS analysis of the headspace of the produced beer samples in the duplicate 2L scale fermentations. (a) Heatmap depicting the relative composition of the volatile metabolomes of the beers. The heatmap is coloured based on Z-scores of the chromatographic peak areas for each compound (normalised to the internal standards 3-octanol and 3,4-dimethylphenol; blue: negative Z-score, red: positive Z-scores. (b,c) Principal component analysis of the same beer volatile metabolomes.Beer 1A&B unhopped control beer, beer 3A&B hopped control beer, Beer 2A&B adjusted unhopped GMO OmANT beer, and Beer 4A&B adjusted hopped GMO OmANT beer.


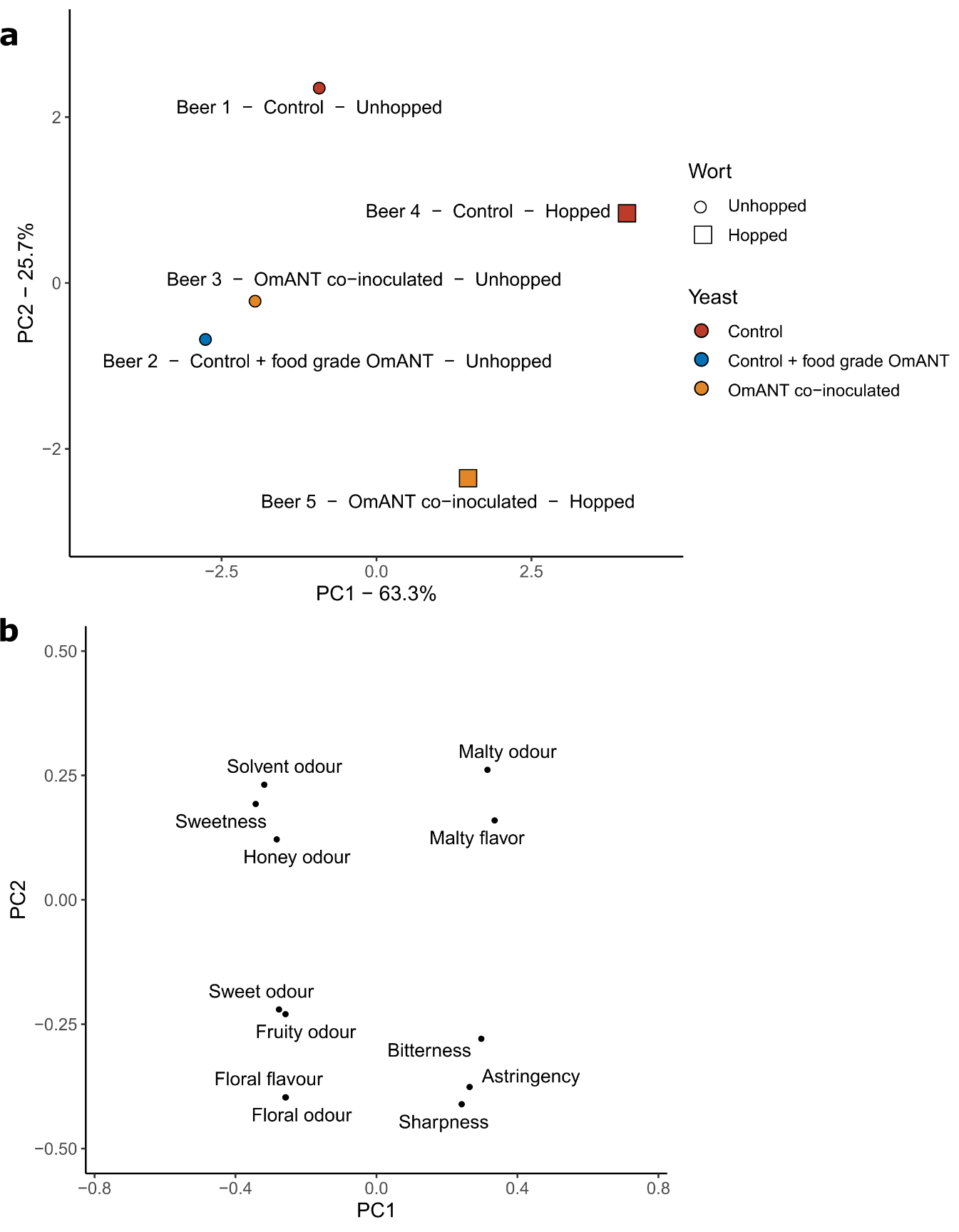


**Figure S**2. PCA biplot of the sensory profiling of the five beers.
